# Grand Rapids Veterinary College

**Published:** 1901-04

**Authors:** 


### GRAND RAPIDS VETERINARY COLLEGE.

THE fourth annual commencement exercises of the Grand Rapids Veterinary College were held in the College Auditorium, Thursday evening, March 28th.

Prof. J. B. Griswold, president of the faculty, who presided over the department of surgical pathology, was master of ceremonies. The hall was beautifully decorated with bunting, potted palms, and ferns. The rostrum was draped with American flags and furnished with elegant leather upholstered furniture. Wurzburg's orchestra furnished music for the occasion.

The valedictory was given by Dr. Henry Haynes, of Jackson, Mich.

The degree of Doctor of Veterinary Science was conferred upon the following gentlemen: Edmund Burke, Calcutta, India; B. Frank Baldwin, Rockford, Mich.; Eugene F. Brown, Lawrence, Mich.; Charles E. Hessey, Glenn, Mich.; Henry Haynes, Jackson, Mich.; George M. Olds, Hartford, Mich.; Charles H. Olds, Grand Rapids, Mich.; A. Z. Nichols, Ransom, Mich.; A. B. Werrener, Portsmouth, Ohio; Simon A. Welch, Grand Rapids, Mich.; William C. Wootten, Grand Rapids, Mich.; Elijah E. Patterson, Detroit, Mich.; R. B. Parker, Hebron, Wis.

The dean, Prof. L. L. Conkey, in his closing address, went over the work of the college since its organization, showing that the whole number of matriculates to date were seventy-two; out of this number forty-two had received degrees, thirty-three receiving the degree of Doctor of Veterinary Science, and nine the degree of Master Farrier.

After the exercises were over the graduating class and faculty, with invited guests numbering fifty persons, went to the Chapin dining parlors, where a bountiful spread awaited them, that had been prepared by the college. Col. M. A. Aldrich was toastmaster. Eber Rice, Drs. Bingham, Innes, Bessey, and Conkey were the local speakers; Dr. Smith, of Coopersville; Dr. P. Hesseltine, of Flint, and Dr. W. B. Thorburn, of Lansing, each responded to a toast. Dr. B. O. Johnson, of Benton Harbor, sent his congratulations to the class, but was unable to be present on account of sickness in his family. Dr. Nelson, of Manistee; Representative Adams, of Van Buren, and Dr. G. H. Stevenson, of Bay City, were also unable to be present.

After the banquet steps were taken to organize a State Veterinary Association, to be known as the Wolverine Veterinary Medical Association, and twenty-one veterinarians signed the articles of association. A charter will be secured in a few days. It was decided to hold the first summer meeting in Grand Rapids, July 3d and 4th next.

